# Training the healthcare workforce to support task-shifting and viral hepatitis elimination: a global review of English language online trainings and in-person workshops for management of hepatitis B and C infection

**DOI:** 10.1186/s12913-023-09777-x

**Published:** 2023-08-11

**Authors:** Maria A. Corcorran, John D. Scott, Marcelo Naveira, Philippa Easterbrook

**Affiliations:** 1 Division of Allergy and Infectious Diseases, Department of Medicine, University of Washington School of Medicine, 325 9Th Ave, Box 359782, Seattle, WA 98104 USA; 2 World Health Organization Regional Office for Europe, Copenhagen, Denmark; 3 Department of Global HIV, Hepatitis and STI Programmes, World Health Organization, Geneva, Switzerland

**Keywords:** Hepatitis B, Hepatitis C, Viral hepatitis elimination, Healthcare worker training

## Abstract

**Background:**

Achieving World Health Organization (WHO) targets for viral hepatitis elimination will require simplification and decentralisation of care, supported through task-shifting and training of non-specialist frontline healthcare workers. To inform development of national health worker trainings in viral hepatitis, we review and summarise available online and workshop trainings for management of hepatitis B virus (HBV) and hepatitis C virus (HCV).

**Methods:**

We performed a systematic search of PubMed, Embase, Web of Science, conference abstracts, and grey literature using Google to identify online and in-person workshop trainings for health workers focused on HBV and/or HCV. Additional trainings were identified through a WHO regional network. We included online trainings written in English and in-person workshops developed for low-and-middle-income countries (LMICs). Available curricula are summarised together with key operational features (e.g. training length, year developed/updated, developing institution) and programmatic features (e.g. content, mechanism for self-assessment, use of clinical case studies).

**Results:**

A total of 30 trainings met our inclusion criteria (10 online trainings; 20 in-person workshops). 50% covered both HBV and HCV, 13% HBV alone and 37% HCV alone. Among online trainings, only 2 (20%) were specifically developed or adapted for LMICs; 70% covered all aspects of hepatitis care, including prevention, assessment, and treatment; 9 (90%) included guidance on when to refer to specialists, and 6 (60%) included modules on management in specific populations (e.g., people who inject drugs [PWID], prisoners, and children). Online trainings used different formats including text-based modules, narrated slide-sets, and interactive web-based modules. Most workshops (95%) were targeted towards non-specialty providers, and 50% were an integral part of a national strategy for viral hepatitis elimination. Workshop length ranged from several hours to multiple sessions over the course of months, and many were part of a blended educational model, which included other opportunities for ongoing learning (e.g., telementorship).

**Conclusion:**

This compendium of online and in-person workshop trainings for HBV and HCV is a useful resource for national hepatitis programmes developing training curricula for non-specialists. Additional online training curricula are needed for use in LMICs, and additional materials are needed to address management challenges in key populations, such as PWID.

## Background

Hepatitis B virus (HBV) and hepatitis C virus (HCV) are leading causes of morbidity and mortality worldwide, especially in low and middle income countries (LMICs) [1, 2]. In 2016, the World Health Organization (WHO) outlined ambitious targets to achieve global elimination of HBV and HCV by 2030, defined as a 90% reduction in new infections and a 65% reduction in mortality. This is achievable through scale-up of testing and treatment, with a goal of diagnosing 90% of those infected and treating 80% of eligible persons, alongside other key preventative interventions including HBV vaccination and harm reduction measures [3, 4]. As of December 2019, it was estimated that 58 million (95% CI 46–76 million) people were chronically infected with HCV, of which only 21% had been diagnosed and 13% treated; and there were an estimated 296 million (95% CI 228–423 million) persons living with chronic HBV infection, of which 10% had been diagnosed and 2% treated. [1]. Achieving the substantial scale-up in testing and treatment required to meet elimination targets will necessitate progressive simplification of care pathways that includes decentralisation of services to primary care, as well as delegation of care to non-specialists through task shifting [5].

Task-shifting or task-sharing of care and treatment to non-specialists was widely adopted as an approach to expand workforce capacity in the global HIV public health response and had a transformative impact on promoting scale-up of antiretroviral treatment globally [6–9]. HBV, which requires similar long-term treatment and monitoring to ensure viral suppression, has the potential to adopt a similar differentiated care model as for HIV, including task-shifting. There are even greater opportunities for task-shifting with HCV, as short course curative treatment requires minimal expertise and monitoring [10–16]. However, until recently, viral hepatitis management has been largely delivered by specialist hepatologists, and there had been relatively little engagement or reported experience from non-specialists or staff from primary care settings [15, 17–19]. A recent large systematic review of both decentralisation and task-shifting to non-specialist health workers in HCV care demonstrated increased uptake of viral load testing and treatment with fully decentralised care and comparable cure rates when care was delivered by non-specialists as compared to specialists [20]. In updated 2022 guidance, WHO now recommends decentralisation of HCV testing, care and treatment, with integration of HCV into existing care services at peripheral health or community-based facilities. These facilities may include primary care, harm reduction sites, prisons, and HIV/ART clinics as well as community-based organizations and outreach services. In order to support decentralisation, WHO also recommends task-sharing by trained non-specialist doctors and nurses to expand access to diagnosis, care and treatment [21, 22].

Nevertheless, despite now widespread access to simplified low-cost direct acting antiviral (DAA) treatment regimens for HCV and potent oral antivirals for HBV, knowledge of viral hepatitis screening and management remains low among frontline healthcare workers and represents a barrier to large-scale decentralisation of care [23–30]. In response to the need for increased provider knowledge of HBV and HCV management to support task-shifting and sharing, our objective was to systematically identify, collate and summarise available resources, materials, and curricula for healthcare worker training that address both initial training and ongoing mentorship. This paper focusses on resources for initial training in HBV and HCV care, through either online training or in-person workshops, while a companion paper evaluates the global experience to date with telementorship for ongoing support of hepatitis care delivery by non-specialists [31].

## Methods

### Study framework

**Fig. 1:**
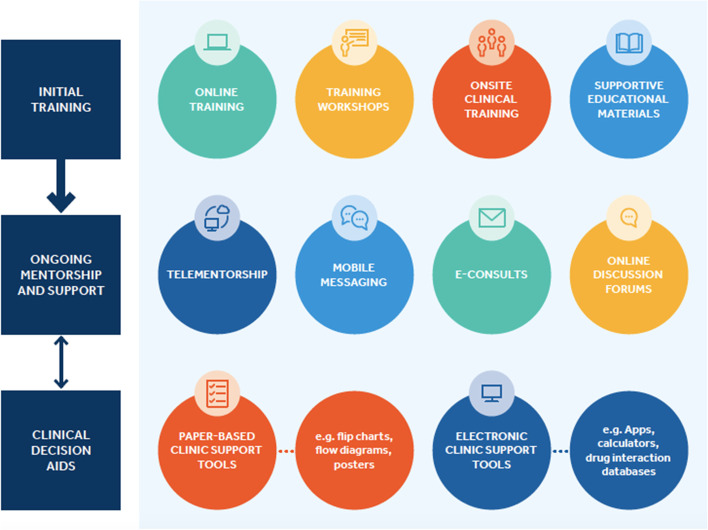
The viral hepatitis training and mentorship toolbox

There are two distinct phases in training the healthcare workforce: 1) initial training and; 2) ongoing mentorship, with additional access to other desktop and online resources (Fig. 1). For initial training, we identified four specific modalities of providing training: online training, in-person workshops, onsite clinical training, and additional training materials such as clinical decision aids, simplified treatment algorithms, test interpretations guides, online and mobile clinical calculators, drug-drug interaction databases, and mobile apps [32].

### Search strategy, selection criteria, and data abstraction

To identify current online trainings and workshops for HBV and HCV management, we performed a comprehensive search of three bibliographic databases (PubMed, EmBase, and Web of Science) following PRISMA guidelines [33]. The search included manuscripts and abstracts published between December 5, 2014, and December 6, 2021. Search terms to identify online trainings and in-person workshops were tailored to the functionality of each database, but generally utilized the following: “hepatitis health worker education,” “hepatitis provider education,” “hepatitis online training,” “hepatitis onsite training,” “hepatitis e-learning,” “hepatitis distance learning,” and “hepatitis didactic.” In addition, accepted conference abstracts (either poster or oral presentations) from the 2019–2021 Asian Pacific Association for Study of the Liver (APASL) and European Association for the Study of the Liver (EASL) that were classified under the category of “viral hepatitis,” “hepatitis B,” or “hepatitis C” were reviewed. A secondary search on Google Web was performed to identify existing literature published outside of traditional journals or conference proceedings for both online trainings and in-person workshops utilizing the search terms “hepatitis training,” “hepatitis health worker education,” and “hepatitis provider education”. The search was limited to websites available in English, and the first 10 pages of Google search results were reviewed for each search term. In addition, a WHO collaborative group network and WHO regional office hepatitis focal points were used to identify other online trainings and viral hepatitis workshops that may have been missed by our bibliographic database, conference abstract and Google searches.

### Selection criteria

Online trainings were defined as educational curricula, available through a website, that could be self-administered without the need to attend an in-person course. Workshops were defined as educational curricula taught by an instructor to learners face-to-face over the course of hours to days. Online trainings were included if their content was published in English and addressed HBV and/or HCV, and if the target audience was healthcare workers, including community-based staff and those working with populations at high risk for viral hepatitis (e.g., harm reduction sites including syringe exchange or opiate substitution therapy programmes). Online trainings targeting only patients, family members, peers, or the general public were excluded. Similarly, online trainings that included a series of stand-alone webinars or slide sets without an underlying unifying curriculum, those where membership in a professional society or payment was required, those developed prior to 2014 (a proxy for the pre-DAA era), and those where slide decks or information had been uploaded from a primary in-person workshop were excluded.

Workshops were included if they were developed for LMICs, targeted healthcare workers, and focused on HBV and/or HCV. Workshops from high-income countries, those developed prior to 2014, and those targeting patients, non-medical personnel, or the general public were excluded. Similarly, curricula from health science schools (e.g., undergraduate medical, pharmacy, or nursing schools) were excluded.

### Data extraction

Data on the following variables was extracted from an in-depth review of each online training or workshop: organization and collaborating partners, target audience, year developed or updated, date of last update (online trainings), training content and curricula (online trainings and workshops as available), whether HBV and/or HCV was covered, and estimated time for completion of each online module or duration of workshop. For online curricula, the following information was also collected: the number of modules and whether all steps on the continuum of care were covered from prevention and testing to treatment and cure, inclusion of modules on specific affected populations (e.g. people who inject drugs [PWID], prisoners, men who have sex with men [MSM], and children), or specific topics (e.g. testing, management of advanced liver disease, triage and referral), and use of clinical case studies, self-assessment tests, and option for continuing education credits. The web address for each training was also recorded. In addition, for workshops, we evaluated whether the workshop was part of a larger work force capacity building initiative at the national level.

### Data analysis

Descriptive statistics were calculated for demographic and operational aspects of online trainings and workshops. Due to limited access to primary training materials for workshops, a narrative review of workshop curriculum was performed for available training content. A series of key best practices were identified for online trainings. The findings were reported in a tabular summary for comparison and included: provision of regular updates, specific modules on management in key populations (PWID, prisoners, children), and on management of advanced liver disease, information relevant to non-specialists such as when to seek help or refer for specialist care, information covering the entire continuum of HBV and HCV care, mechanism for self-assessment, and use of clinical case studies. All data analysed during this study are included in this published article.

## Results

### Online training and workshop search and study selection

**Fig. 2:**
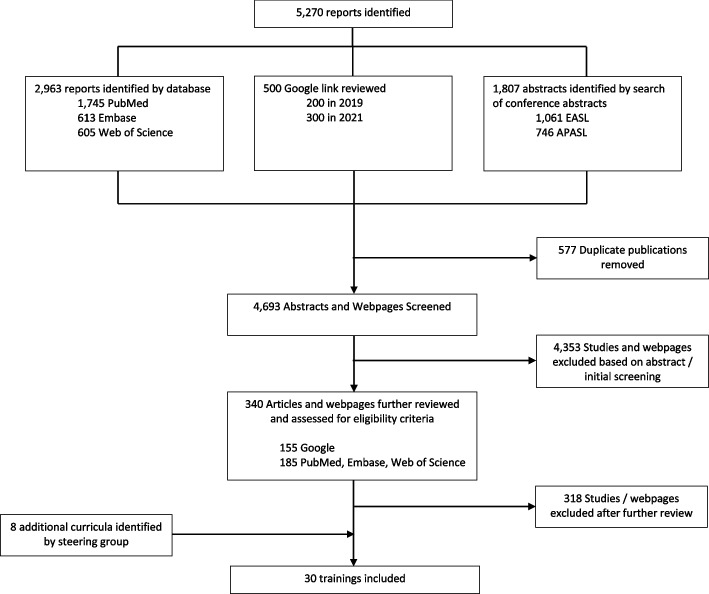
Identification and selection of online trainings and workshops

Figure 2 summarises the flow chart for identification and selection of eligible online trainings and in-person workshops. We identified 5,270 potentially relevant results. After removing duplicates, 4,693 abstracts and webpages were screened, of which 4,353 were excluded. A total of 340 articles and webpages from our bibliographical and online searches were further assessed for eligibility criteria, of which 318 did not meet inclusion criteria or reported on a duplicate curriculum. An additional 8 curricula were identified through our WHO collaborative group network and WHO regional office hepatitis focal points. In total, we identified 30 viral hepatitis trainings meeting inclusion criteria, 10 online curricula and 20 in-person workshops.

### Online trainings

#### Key programmatic and operational features of 10 online trainings

##### Overview

Table 1 summarises the characteristics of the ten identified online trainings. Of these 10 trainings, 8 were developed in the WHO region of the Americas [34–41], 1 in the WHO Western Pacific region [42], and 1 in the WHO Africa region [43]. The majority 8 (80%) of online trainings were developed by and designed for use in high-income countries. The exceptions were the International Association of Providers of AIDS Care (IAPAC) [43] training which had developed materials specifically for use by countries or healthcare providers in Sub-Saharan Africa and Stanford University’s Asian Liver Center Know HBV/HCV online curriculum [40], which was translated into Vietnamese, Chinese, and Mongolian (in addition to English). The AIDS Education and Training Center’s [AETC] HIV/HCV Co-infection National Curriculum [37] is targeted towards HIV providers, and the Australasian Society for HIV, Viral Hepatitis and Sexual Health Medicine’s (ASHM) HBV s100 Prescribers Course [42] (one of several ASHM courses), is aimed at healthcare providers who wish to become HBV antiviral prescribers. Three (30%) of the online curricula covered both HBV and HCV, with 5 (50%) covering HCV only, and 2 (20%) covering HBV only. In general, trainings ranged from 3 to 15 modules, with the estimated time to complete each module ranging from 30 min to approximately 6 h. Six (60%) online trainings offered free continuing education credits.

**Table 1:** Operational characteristics and curricular features of HBV and HCV online training curricula for healthcare workers

Online Training	Website Address	Key Source Institution, Country	Number of Modules	Covers HBV, HCV or Both	Estimated Time to Complete	Free Continuing Education Credits (Y/N)	Updated in Last 2 years*	Information on Key Populations	Info on mgmt. of advanced liver disease	Info on when to refer to higher level of care	Curriculum covers continuum of care	Self-assessment	Use of clinical vignettes
**Hepatitis C Online**	https://www.hepatitisc.uw.edu/	University of Washington, USA	6	HCV	2-6 h per module	Yes	√	√ (PWID, prisoners)	√	√	√	√	
**Hepatitis B Online**	https://hepatitisb.uw.edu/	University of Washington, USA	9	HBV	30 min – 2 h	Yes	√			√	√	√	
**Liver Learning: Fundamentals of Liver Disease:** • **Hepatitis C 2.0** • **Hepatitis B 2.0**	https://liverlearning.aasld.org/	AASLD, USA	15	HBV, HCV	30-40 min each	Yes	√	√ (peds, separate webinar for treatment in PWID)	√	√	√	√	
**ASHM/INHSU**	lms.ashm.org.au/ https://www.inhsu.org/online-learning-modules/	Australasian Society for HIV, Viral Hepatitis and Sexual Health Medicine, Australia International Network on Health and Hepatitis in Substance Users	Variable depending on the training. Max 9	HBV, HCV	Variable	Yes	√	√ (PWID)	√	√	√	√	√
**HIV/HCV Co-infection: An AETC National Curriculum**	Aidsetc.org/hivhcv	AIDS Education and Training Center (AETC), USA	6	HCV, HCV/HIV	1-2 h per module	Yes	√	√ (HIV + , racial minorities)		√	√	√	
**IAPAC** • **HBV Clinical Management** • **HCV Clinical Management**	iapac.org/education/african-regional-capacity-building-hub/	International Association of Providers of AIDS Care (IAPAC), South Africa	11	HBV, HCV	1 day per module	No				√	√	√	√
**CATIE** • **Hepatitis C Basics** • **Hepatitis C Treatment**	https://www.catie.ca/education-publications-websites-education/self-directed-learning-0	Canadian AIDS Treatment Information Exchange, Canada	6	HCV	1 h per module	No		√ (PWID)		√	√	√	√
**Health E Knowledge**	http://healtheknowledge.org/course/view.php?id=100	Addiction Technology Transfer Center Network, USA	4	HCV	90 min total	Yes				√		√	√
**Know HBV and HCV**	https://www.edx.org/course/know-hbv-and-hcv?index=product&queryID=5a5a485a457dafdb2b041b7f0eb3427f&position=1	Stanford University, USA	3	HBV	3 h	No	√					√	
**British Columbia Center for Disease Control: Hepatitis C Course for Public Health Providers**	https://hepatitiseducation.med.ubc.ca/providers/	University of British Columbia, Canada	6	HCV	3-4 h	No	√	√ (PWID, indigenous populations)		√		√	

*As of 2021

#### Key features of 10 online trainings

##### Scope of modules

Key features of curricula for individual online trainings are outlined in Table 1. The majority of online curricula (70%) covered the full continuum of HBV and/or HCV care from prevention, screening, treatment and long-term monitoring (HBV) or cure (HCV), and screening for liver cancer; 9 (90%) also included guidance on when to consult and refer for specialist care, and 6 (60%) had modules on management in specific populations (e.g. PWID, prisoners, children, indigenous populations), although the level of detail included regarding management of key populations was variable across trainings. Only three (30%) contained specific information on the management of advanced liver disease.

##### Curriculum

Common topics/modules covered by all trainings were the epidemiology, prevention, and natural history of HBV and/or HCV, evaluation and preparation for treatment including fibrosis staging, and HBV and/or HCV treatment and regimens (Tables 2 and 3). Trainings targeted more specifically at healthcare workers and prescribers [34–36, 42, 43] provided more extensive information on viral hepatitis treatment. Management of viral hepatitis in specific populations was captured well in 6 trainings: the AASLD Liver Learning curricula for HBV and HCV module on treatment of HBV and HCV in children [36]; the AETC HIV/HCV co-infection curriculum module on barriers to care for co-infected persons of color [37]; and University of Washington’s Hepatitis C Online curriculum modules on HCV treatment in persons with substance use disorders and in prison settings [34]; the ASHM/International Network on Health and Hepatitis in Substance users (INHSU) online HCV training specifically for needle and syringe programme frontline workers focusing on HCV [42]; the CATIE curriculum [38], which included information on PWID; and the BCCDC Hepatitis C Course for Public Health Providers [41], which included information on PWID and indigenous populations.

**Table 2:** Common curricular elements for hepatitis C trainings

COMMON CURRICULAR ELEMENTS: HCV HEALTHCARE WORKER TRAININGS
**1.** **Epidemiology and prevention**
**2.** **Natural history**
**3.** **Screening and diagnosis**
**4.** **Evaluation and staging of liver fibrosis, preparing for treatment**
**5.** **Treatment of chronic hepatitis C, including monitoring and follow up**
**6.** **Management of advanced liver disease and cirrhosis and/or when to refer to a higher level of care**
**7.** **HCV management in key populations and those with co-morbidities (e.g. HIV coinfection, renal failure, PWID)**

**Table 3:** Common curricular elements for hepatitis B trainings

COMMON CURRICULAR ELEMENTS: HBV HEALTHCARE WORKER TRAININGS
1. **Epidemiology and prevention, including information on occupational exposure and immunizations**
2. **Natural history and virology**
3. **Screening and diagnosis, including interpretation of serologic markers**
4. **Evaluation and staging of liver fibrosis**
5. **Identification of treatment candidates and treatment of chronic HBV infection**
6. **Management of advanced liver disease and cirrhosis and/or when to refer to a higher level of care**
7. **HBV management in key populations and those with co-morbidities (e.g. pregnant women, HIV, HCV or HDV coinfection, renal failure, children)**

##### Presentation format

A range of presentation formats and features were used to promote the accessibility and usability of content for healthcare worker learning. These included: easy to navigate text-based modules; narrated slide sets; interactive web-based modules; and links to advanced learning tools and other resources, as summarised below.

1. **Easy to navigate text-based modules:** Two of the online trainings, Hepatitis C Online and Hepatitis B Online [34, 35], adopted text-based modules, sub-divided into different topic pages, allowing the learner to advance through topics at their own pace with options to skip or selectively review sections. Illustrative figures were used to reinforce learning points with a search function for easy navigation.
2. **Narrated slide sets**: Several trainings were presented in a narrated slide set format. One example is the AASLD’s Liver Learning Fundamentals of Liver Disease for HBV and HCV [36]. The AASLD HBV and HCV programmes follow a clear curriculum with video modules of approximately 30–40 min in length. Slide decks are narrated by different experts in the field, with a 5-question pre and post-test before and after each module.
3. **Interactive web-based modules**: The CATIE Hepatitis C Basic’s curriculum [38] and the ASHM/INHSU training on Curing Hepatitis C in Primary Care [42] are both presented in an interactive learning format with a set of clinical case-based scenarios each and questions the learner must answer to progress through the module, with correct answers justifications provided.
4. **Other features**:
  - **Links to other online educational and learning tools and other resources related to liver health:** AASLD’s online modules on HBV and HCV [36] represent just two curricula in a larger “Fundamentals of Liver Disease” series, which includes relevant general modules on cirrhosis, interpretation of abnormal liver tests, and non-alcoholic fatty liver disease. The AASLD “Liver Learning” online platform also contains links to multiple webinars on select topics in liver disease and recordings of prior AASLD conference sessions, and the AASLD guidelines. The University of Washington’s Hepatitis C Online [34] contains additional interactive sections on HCV biology (with graphical overview of HCV structure, proteins and lifecycle), HCV medications and tools (slide decks reviewing clinical trial data for approved HCV drugs) and online calculators for scores such as APRI and FIB-4. A master bibliography is also included.
  - **Link to telephone or web-based consultation:** Hepatitis C Online [34] includes a link to the University of California San Francisco’s Clinical Consultation Center, which offers provider to provider telephone and web-based consultation for HCV, HIV, and substance use disorders.

##### Pre and post-test, end of module quiz or interactive self-assessment test

All trainings offered some form of self-assessment, usually as multiple-choice questions with feedback at the end of each topic / module and/or a pre and post course quiz.

##### Provision of materials in other languages

ASHM has partnered with several other organizations such as the International Networks on Hepatitis in Substance Users (INSHU), the University of New South Wales, and the Kirby Institute to make its materials available in several languages, including German, Dutch, French, Italian, Portuguese, Swedish, and Spanish, via the INSHU website [44]. Similarly, Stanford University’s Know HBV training [40], presented in a narrated video slide deck format, has been developed in English, Vietnamese, Chinese and Mongolian.

##### Programme evaluation

There are limited data on programme metrics and evaluation of online trainings. Of the 10 identified online trainings, only two had a published programme evaluation [45, 46]. The Asian Liver Center at Stanford University published an evaluation of the implementation of an early version of their Know HBV/HCV course in Shandong Province, China in 2011 among 1015 health professionals [45]. In this report they observed that the proportion of correct answers increased from 19.6% on a pre-test to 42.4% on a post-test (p-value < 0.001). Ninety-four percent of participants also reported that the instructional approach was helpful, and 93.2% preferred the online format over traditional classroom-based learning [45]. The AIDS Education and Training Center (AETC) similarly evaluated their curriculum using pre-and post-test scores and showed short term knowledge gain through their HIV/HCV co-infection curriculum [46].

### Workshops

#### Key programmatic and operational features of 20 in-person workshops

Table 4 summarises key features of identified workshops. Of the 20 workshops identified, 6 were delivered in the WHO Africa region, 4 in the WHO Eastern Mediterranean region, 3 in the WHO Southeast Asia region, 1 in the WHO Western Pacific region, 1 in the WHO Region of the Americas, 3 in the WHO Europe region, and 2 in multiple regions. Twelve (60%) of the 20 identified workshops covered both HBV and HCV, with 6 (30%) covering HCV only and 2 (10%) covering HBV only. The workshop durations ranged from 2–3 h to 8 sessions over the course of 10 months. All workshops were targeted towards non-specialty providers with the exception of the Egyptian “train the trainer” workshops, targeted at updating specialists on key changes in guideline recommendations or other aspects of clinical management for persons with chronic HCV.

**Table 4:** HBV and HCV workshops for healthcare workers in low-and-middle-income countries

Workshop	Curriculum Developer	Collaborating Partners	Year Developed	Duration of Training	Covers HCV, HBV, or Both	Target Audience	Part of a National Program / Strategic Plan for Viral Hepatitis
WHO SEARO/WPRO	WHO Southeast Asian and Western Pacific Regional Offices	TREAT Asia	2018	3 days	HBV and HCV	Primary care, ID and HIV physicians in the WHO SEARO and WPRO regions	No
Nigerian National Hepatitis Control Program	Nigerian Ministry of Health	Clinton Health Access Initiative^a^	2017	2 days	HBV and HCV	Primary healthcare providers in Nigeria	Yes
Muhk Mantri Pujab Hepatitis C Relief Fund	Punjab State Government	Post Graduate Institute of Medical Education and Research, Chandigargh	2016	4-6 h	HCV	Internal medicine physicians at 22 district hospitals and 3 government hospitals	Yes
Rwanda Viral Hepatitis Curriculum	Rwandan Ministry of Health	Duke University, Clinton Health Access Initiative^a^	2015	1wk	HCV	General practitioners and nurse specialists	Yes
Rwanda ASCEND Model	University of Maryland	Rwanda Biomedical Center, Maryland Global Initiative Corporation	2016	8 2-3 h session over 10 months	HCV	Physicians in Kigali, Rwanda who had never treated HCV but had HIV experience	No
Myanmar Viral Hepatitis Symposium	B.K. Kee Foundation, Stanford University, Myanmar Ministry of Health and Sports		2016	2 days	HBV and HCV	Primary care and specialty physicians across Myanmar	Yes
Institute for Liver and Biliary Sciences, New Delhi India	Collaborative groups of physicians, microbiologists and public health personnel at ILBS	ILBS is a WHO collaborating partner	2015	1 day	HBV and HCV	Different curricula for primary care physicians, nurses, and lab technicians at community and primary health centers	No
Egypt Train the Trainer Workshops	Egyptian National HCV Committee			1 day	HCV	Specialist providers in Egypt who work at either academic centers or liver specialty clinics	Yes
Pakistan Kidney and Liver Institute	Pakistan Kidney and Liver Institute		2017	2 weeks	HBV and HCV	General practitioners, nurses, pharmacists and lab technicians at 24 clinics sites in Lahore Province	Yes
Mongolia Viral Hepatitis Symposium	Mongolia Ministry of Health and Sports	Stanford University	2015	2 days	HBV and HCV	Primary care and specialty physicians treating or planning to treat viral hepatitis in Mongolia	Yes
Tanzania Healthcare Worker HBV Education	University of Minnesota	Ilula Lutheran Hospital	2020	≤ 1 day	HBV	Community healthcare workers	No
Comprehensive Countermeasures for Viral Hepatitis	National Hospital Organization Kumamoto Medical Center	Japan International Cooperation Agency	2015^b^	Not known	HBV and HCV	Healthcare workers in LMICs, including Egypt	No
Iranian Hepatitis Network (IHN) World Hepatitis Day Workshop	Iranian Hepatitis Network		2016	≤ 1 day	HBV and HCV	Health sciences students	No
Tanzania HBV Demonstration Project		U.S. Centers for Disease Control and Prevention; Gilead Sciences	2016		HBV	Healthcare workers and community partners in Dar es Salaam and Zanzibar	Yes
WHO Africa Region	WHO Regional Office for Africa	WHO Southeast Asian and Western Pacific Regional Offices	2022	1 day to multiple days, adaptable to context	HBV and HCV	Healthcare workers in the WHO Africa Region	No
The Training in Emerging Advances in the Management of Chronic Hepatitis For Health Care Professionals in Pakistan (Teach Pak Program)	Pakistan Society for the Study of Liver Disease (PSSLD)	Multiple, including Aga Khan University & Hospital	2012, made into a certificate course in hepatology in 2016	1 day a week for 4 weeks	HBV and HCV	Medical students, post-graduate trainees, family physicians	No
Médecins Sans Frontièrs (Ukraine)	Médecins Sans Frontièrs	КHП "Mикoлaївcький oблacний цeнтp пaлiaтивнoї дoпoмoги тa iнтeгpoвaниx пocлyг" MOP	2017	2 days	HBV and HCV	Healthcare workers	No
Uzbekistan Hepatitis Elimination Project (UHEP)	CDA Foundation	Uzbekistan Research Institute of Virology University of Maryland	2019	4 h	HBV and HCV	General practitioners	Yes
International Training & Education Center for Health (Ukraine)	International Training & Education Center for Health (I-TECH)	Chas Zhyttia Plus Center for Public Health of the Ministry of Health of Ukraine	2015, 2017	5 days	HCV (and HIV)	Healthcare workers	No
Brazil Ministry of Health HCV Training	Brazil Ministry of Health	Department of Pharmaceutical Assistance of the Secretariat of Science, Technology and Strategic Inputs Brazilian Health Regulatory Agency (Avisa)	2015	2–3 h	HCV	Healthcare workers	Yes

^a^The Clinton Health Access Initiative (CHAI) has provided technical assistance to Cambodia, India, Indonesia, Myanmar, Nigeria, Rwanda and Vietnam, with over 5,900 healthcare workers trained collectively

^b^Viral hepatitis course initially debuted in 1998, but was renewed as the “Comprehensive Countermeasure for Viral Hepatitis” in 2015

Ten (50%) of the workshops were an integral part of a national strategy for viral hepatitis elimination and health workforce capacity building and received direct support from the local or national ministry of health. Many workshops also benefited from strong collaborations with non-governmental organizations or outside universities. For example, the Clinton Health Access Initiative (CHAI), collaborated with both the Nigerian and Rwandan ministries of health to develop national training for primary healthcare providers [47], and also provides technical assistance to Cambodia, India, Indonesia, Myanmar, and Vietnam, with more than 5,900 healthcare workers trained across these 7 countries [48]. Similarly, Stanford University collaborated with both the Myanmar Ministry of Health and Sports and the Mongolia Ministry of Health and Sports to support development of a two-day viral hepatitis training symposiums in each country [26, 49].

##### Curriculum

In contrast to online trainings, an in-depth review of the primary curriculum for each identified workshop was not possible. Published data on certain workshops trainings [26, 47, 49–56] indicate that the curricula were generally comprehensive, covering epidemiology, diagnosis, workup, and treatment of HBV, HCV, or both. We highlight two specific trainings, the WHO Southeast Asia and Western Pacific regional training [57] and the Institute of Liver and Biliary Sciences “Hepatitis Induction Program” for nurses [58] Tables 5, 6, 7, 8 and 9.

**Table 5:**
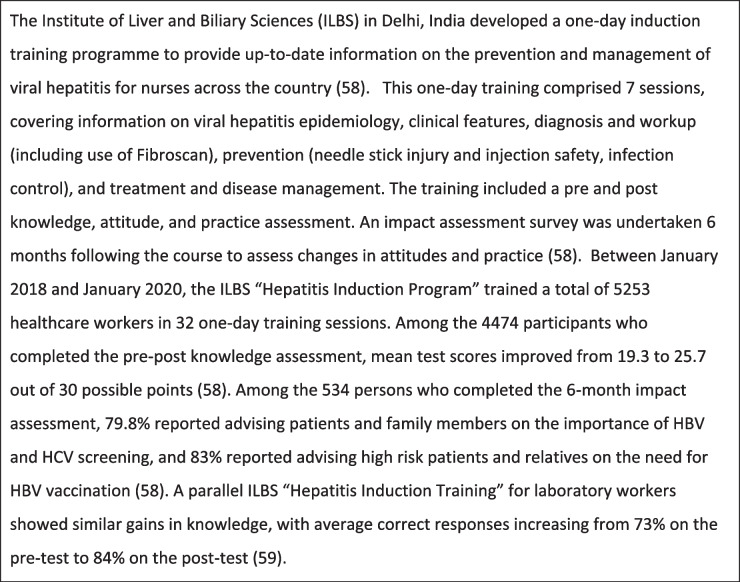
India: Institute of Liver and Biliary Sciences “Hepatitis Induction Program” for nurses [58, 59]

**Table 6:**
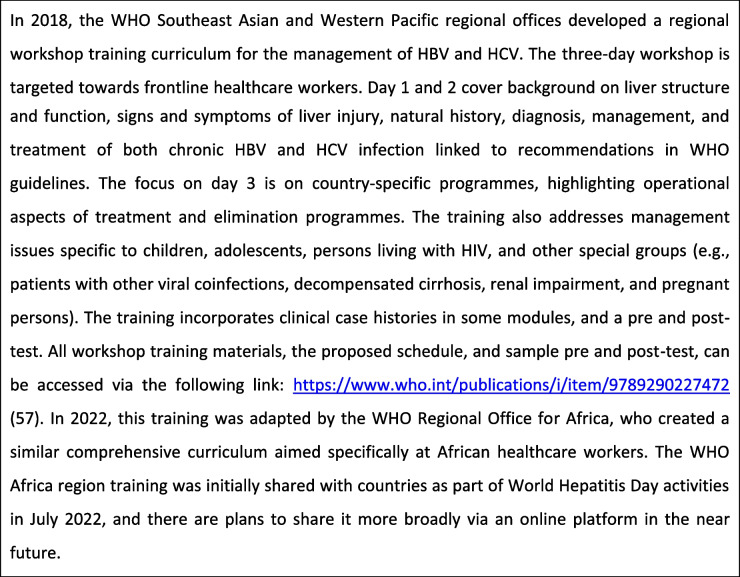
WHO Southeast Asia and Western Pacific regional training in viral hepatitis [57]

##### Blended learning models

Several of the 20 identified workshops were developed as one component or as an option alongside other opportunities for ongoing learning and clinical support, such as telementorship, clinical attachments, and WhatsApp groups (Table 7).

**Table 7:**
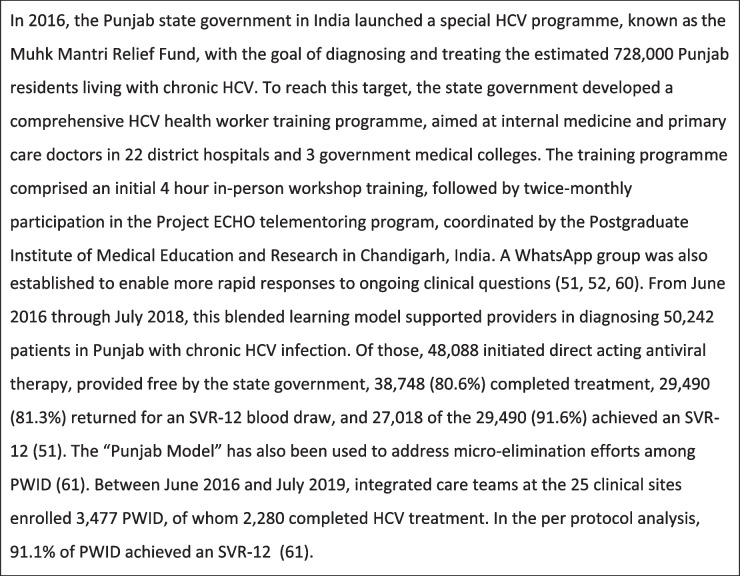
Punjab, India: HCV blended training programme [51, 52, 60, 61]

##### Programme evaluation

In comparison to online trainings, there has been more published evaluations of viral hepatitis workshop trainings, both based on programme metrics for blended learning programmes reported above and published reports from workshops and educational initiatives in countries including Rwanda, Myanmar, Mongolia, Egypt, Iran, and Tanzania [26, 47, 49, 53–56, 62, 63] (Tables 8 and 9).

**Table 8:**
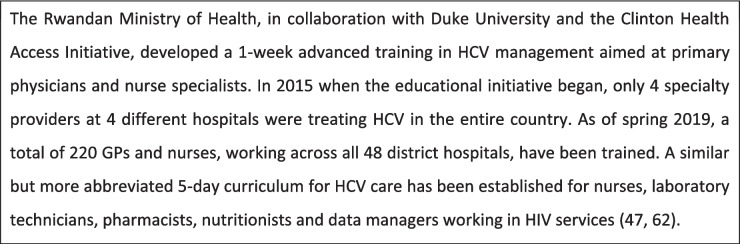
Rwandan Ministry of Health advanced training in HCV management [47, 62]

**Table 9:**
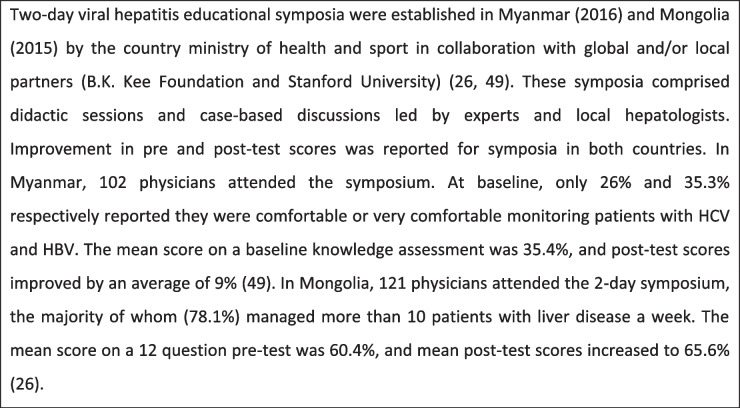
Myanmar and Mongolia: Viral hepatitis symposia [26, 49]

#### Other Training Resources for Healthcare Workers:

In addition to online curricula and in-person workshops for healthcare worker training, Table 10 lists examples of other resources available to support healthcare worker education and decision making. These include both paper-based clinical support tools (e.g., flip charts, flow diagrams, posters) and electronic clinical support tools (e.g., mobile apps, clinical calculators, drug-drug interaction databases).

**Table 10:** Additional resources to support HBV and HCV decision making at the point of care

Additional Resources for Healthcare Workers	Examples
1. **Practice support toolkits** ◦Basic information on viral hepatitis (e.g., HBV and/or HCV basics) ◦ Patient support resources (e.g., patient support organizations, health promotion resources) ◦ Provider support resources (e.g., educational opportunities, how to access specialists support) ◦ Practice support resources (e.g., billing support, tracking progress)	**Eliminate Hepatitis C Partnership Practice Support Toolkit (Australia)** https://ecpartnership.org.au/toolkit
2. **Mobile apps to support treatment decision making** ◦ Guideline-based mobile algorithms to assist with treatment decision making, particularly when starting a new therapy	**Know HBV: Chronic Hepatitis B Treatment Decision Tool for Adults** https://nohepb.org/#/
3. **Drug-drug interaction databases** ◦ Online and mobile drug interaction databases to help ensure the safe co-administration of medications during viral hepatitis treatment	**University of Liverpool’s Hepatitis Drug Interaction Database** https://www.hep-druginteractions.org/checker
4. **Clinical calculators** ◦ Available online and via mobile apps for assistance in calculating scores such as APRI, Fib-4, and MELD	**University of Washington’s Hepatitis C Online: Tools & Calculators** https://www.hepatitisc.uw.edu/page/clinical-calculators/apri
5. **Paper based clinical support tools** ◦ Printed materials, often found in a clinic setting, that provide guidance on diagnostic pathways, test interpretation, fibrosis staging, and decision making for treatment initiation. Can take various forms including: ▪ Posters ▪ Flip charts ▪ Flow diagrams	**Burnett Institute: ‘Think Hep B’** https://www1.racgp.org.au/RACGP/media/AJGP/documents/Appendices/Supplement-1-Think-Hep-B.png

While this review did not systematically search for or evaluate webinars, examples of sites with viral hepatitis educational content delivered in webinar format include the following:

1. New York State Department of Health AIDS Institute: Clinical Education Initiative (https://ceitraining.org/courses/)
2. European Associations for the Study of the Liver (https://easl.eu/event_type/webinar/)
3. PRIME (https://primeinc.org/cme/credit/infectious-disease/all)
4. HepCure (https://hepcure.org/webinars/)
5. National Alliance of State and Territorial AIDS Directors (https://nastad.org/teams/hepatitis#webinars)
6. European AIDS Treatment Group (https://www.eatgtrainingacademy.com/hepatitis-webinar-recordings

## Discussion

The expansion of national health workforces to support scale-up of HBV and HCV testing, care and treatment to decentralised sites such as primary care and harm reduction sites requires access to effective training materials. In this review, we provide a selected compendium of some of the available English language training resources – both online courses, and in person workshops to help guide delivery of national or facility level training programmes. Although this was not a comprehensive review of all available online trainings or in-person workshops, we consider that it is a fair representation of some of the most widely available and disseminated products. Ten online trainings and 20 workshops met our pre-specified inclusion and quality criteria and provide a useful foundation for countries to adapt for use in the development of their national viral hepatitis training programmes. The majority of these trainings were targeted at primary care or non-specialist health workers and provided a comprehensive curriculum that covered background information on epidemiology, and liver function, as well as management across the continuum of care for viral hepatitis, including screening, initial work-up and staging of disease, treatment, and monitoring for either HCV cure and/or long-term follow-up for HBV. Overall, half of the trainings identified addressed both HCV and HBV, over one-third HCV alone and the remainder HBV alone. We also identified several good practices with both online and workshop training materials to further inform future national training approaches. A combination of these educational approaches forms the basis of a successful viral hepatitis provider toolkit.

We identified key messages regarding the future direction of viral hepatitis training. First, the growing importance of online training. While there have been few published evaluations of online viral hepatitis training, there is growing evidence that learners often prefer the convenience of online learning compared to traditional classroom-based instruction [45], and that ability to illustrate complex topics in a flexible online format may lead to substantial gains in knowledge [46, 64]. This is in keeping with literature in other specialties and allied health professions, where learners report high satisfaction with online learning [65], and where online learning has been shown to be non-inferior to face-to-face instruction [66, 67]. Distinct advantages of online training include the ability to link directly to other training resources, flexibility to convey information in differing user-friendly formats, to provide information in multiple languages, and to provide real-time feedback on knowledge-based questions or self-assessment. Furthermore, online training has emerged as a highly flexible and adaptive approach to meet rapidly changing educational needs in the era of COVID-19 and social distancing. Data evaluating online trainings since the start of the COVID-19 pandemic have supported the acceptability, efficacy, and flexibility of online training, as well as the scalability of well-developed e-learning platforms [68–73].

Second, the strategic use of in person-workshops and blended learning approaches. Although, the future of large in-person training workshops remains unclear, our review identified some advantages of in-person workshops for viral hepatitis education. These include the ability to readily adapt trainings to different regional and country contexts as well as to local priority populations such as PWID or prisoners (e.g. WHO WPRO, SEARO, and Africa region trainings). Similarly, a key success factor identified from viral hepatitis training workshops in Punjab, India and Punjab, Pakistan was the adoption of blended learning approaches of in-person workshops with other educational approaches and support such as telementoring or clinical attachments to create a comprehensive educational programme [51]. Additional good practices include the use of multiple clinical case studies to illustrate key learning points, links to other educational resources – including calculators, and online and in person training materials that are regularly updated.

There are a number of limitations to this descriptive review. Although, we used a range of search strategies to identify both online training sites and in-person workshops on viral hepatitis based on specified inclusion criteria, we recognize that many online trainings and workshops, including several in development, will have been missed. This particularly applies to those where there was little promotion of the course or where the slides were not made available in the public domain. Trainings supported by collaborating partners, where funding was available to disseminate the materials and perform workshop evaluations were more likely to have been identified. The limitation of our search to online curricula available in English will have also excluded many important non-English course materials in other key UN or local languages. The Ministry of Health in Brazil, for example, has developed a 15-h online training for HBV and HCV in Portuguese (https://telelab.aids.gov.br/index.php/component/k2/item/94-diagnostico-de-hepatites-virais), which did not meet our prespecified language inclusion criteria but represents an important resource for healthcare providers in Brazil and other Portuguese speaking nations. The majority of trainings were developed by and used in high income settings with curricula based on North American, Australian, Canadian and European guidelines, with few adapted for use in LMICs. Only one online training was developed exclusively for use in low- and middle-income settings – the International Association of Providers of AIDS Care train the trainer curriculum developed for Sub-Saharan Africa; although it is worth noting that Stanford University’s Know HBV training has been adapted to low-and-middle-income settings, with versions published in Vietnamese, Mongolian and Chinese, in addition to English. Our search may have missed online trainings that had previously been used but recently taken off-line, and our search for online curricula excluded a review of websites, such as PRIME Network or Medscape, that publish online webinars, as these represent single presentations on specific topics in the field. Webinars do, however, represent an important modality for online learning, particularly for healthcare workers who are already active in the field, and as such, we may be excluding a large subsector of the online learning environment. Additionally, the relative lack of published data evaluating online and workshop curricula limits our ability to compare these curricula and training approaches in a more systematic way, as data on participant usage, change in pre and post-test scores, impact on practice and patient level outcomes, and other metrics, are not widely available. Finally, we were not able to systematically collect information on funding for each training, as this information was not consistently available in published materials. Differences in funding likely contribute to variability in the quality of trainings, and funding represents a major consideration when considering scale up of online and workshop trainings.

We identified several opportunities to improve existing online and in-person training models for healthcare worker education, particularly in low-and-middle income countries. First, the availability of a comprehensive regionally adapted online training programme on the management of HBV and HCV in LMICs based on WHO HBV [74] and HCV [5, 21, 22] guidelines or LMIC country-specific guidelines, would provide a strong foundation to support a national training programme, especially given the ongoing COVID social distancing practices. Such an online programme can be blended with in-person workshops, as well as telementorship or mobile messaging groups for ongoing clinical support. Second, although most trainings were comprehensive in coverage of key information relevant to clinical management across the continuum of viral hepatitis care, there were few modules on practical aspects of implementation in specific populations such as PWID, prisoners, or care in children and adolescents. There were also no specific trainings in cultural sensitivities and competencies in addressing stigma, given the common feedback from clients from marginalized groups such as PWID and prisoners of experiencing discrimination from healthcare staff [75–77]. As task-shifting and decentralisation of HBV and HCV care becomes more common, education on the provision of HBV and HCV treatment within non-traditional settings, such as needle and syringe exchange programmes and other harm reduction centers, will also be needed. Nevertheless, while development of trainings focusing on key populations and implementation of viral hepatitis treatment within non-traditional settings represents an opportunity for the future, we also recognize that such development may at times be limited by insufficient epidemiologic data on HBV and HCV prevalence among key populations, particularly in LMICs. Third, it must be recognized that different cadres of healthcare workers will have varying training needs, particularly as decentralisation of HBV and HCV care becomes more common in non-traditional settings, such as at harm reduction sites or in the community. Adaptation of materials will also be needed to reflect availability of different treatment regimens and diagnostics and different systems for funding of care and out of pocket costs for patients. Online training and workshop curricula also need to be considered as part of a larger toolkit of resources available for healthcare worker education (Fig. 1). Finally, there is a need for more evaluation on the comparative impact of these different trainings, on improving workforce knowledge and practical competencies as well as in expanding workforce capacity.

## Conclusions

Online and workshop curricula represent an important component of the viral hepatitis workforce training toolkit to support decentralisation and task-shifting of HBV and HCV care. This compendium of 10 online courses and 20 in person workshops provides a foundation for national programmes to deliver training programmes adapted for viral hepatitis care in LMICs. Finally, increased efforts are needed to evaluate new and existing viral hepatitis healthcare worker trainings to promote the dissemination of good practices.

## Data Availability

All data generated or analysed during this study are included in this published article.
